# Organ-Specific MicroRNAs (*MIR122, 137,* and *206*) Contribute to Tissue Characteristics and Carcinogenesis by Regulating Pyruvate Kinase M1/2 (*PKM*) Expression

**DOI:** 10.3390/ijms19051276

**Published:** 2018-04-24

**Authors:** Kohei Taniguchi, Nobuhiko Sugito, Haruka Shinohara, Yuki Kuranaga, Yosuke Inomata, Kazumasa Komura, Kazuhisa Uchiyama, Yukihiro Akao

**Affiliations:** 1 Department of General and Gastroenterological Surgery, Osaka Medical College, 2-7 Daigaku-Machi, Takatsuki, Osaka 569-8686, Japan; sur171@osaka-med.ac.jp (Y.I.); uchi@osaka-med.ac.jp (K.U.); 2 Translational Research Program, Osaka Medical College, 2-7 Daigaku-Machi, Takatsuki, Osaka 569-8686, Japan; uro051@osaka-med.ac.jp; 3 United Graduate School of Drug Discovery and Medical Information Sciences, Gifu University, 1-1 Yanagido, Gifu 501-1193, Japan; v3501002@edu.gifu-u.ac.jp (N.S.); harukashinohara313@gmail.com (H.S.); v3501001@edu.gifu-u.ac.jp (Y.K.); yakao@gifu-u.ac.jp (Y.A.)

**Keywords:** microRNA, *PKM*, *PKLR*, *PTBP1*, Warburg effect

## Abstract

Pyruvate kinase is known as the glycolytic enzyme catalyzing the final step in glycolysis. In mammals, two different forms of it exist, i.e., pyruvate kinase M1/2 (*PKM*) and pyruvate kinase L/R (*PKLR*). Also, *PKM* has two isoforms, i.e., *PKM1* and *PKM2*. These genes have tissue-specific distribution. Namely, *PKM1* is distributed in high-energy-demanding organs, such as brain and muscle. Also, *PKM2* is distributed in various other organs, such as the colon. On the other hand, *PKLR* is distributed in liver and red blood cells (RBCs). Interestingly, *PKM2* has been recognized as one of the essential genes for the cancer-specific energy metabolism termed the “Warburg effect”. However, the mechanism(s) underlying this fact have remained largely unclear. Recently, we found that some organ-specific microRNAs (miRNAs, *MIR*) regulate *PKM* isoform expression through direct targeting of polypyrimidine tract binding protein 1 (*PTBP1*), which is the splicer responsible for *PKM2*-dominant expression. In this study, we examined whether this machinery was conserved in the case of other *PTBP1*- and *PKM*-targeting miRNAs. We focused on the *MIRs 122*, *137*, and *206*, and investigated the expression profiles of each of these miRNAs in tissues from mouse and human organs. Also, we examined the regulatory mechanisms of *PKM* isoform expression by testing each of these miRNAs in human cancer cell lines. Presently, we found that brain-specific *MIR137* and muscle-specific *MIR206* predominantly induced *PKM1* expression through direct targeting of *PTBP1*. Also, liver-specific *MIR122* suppressed the expression of both *PKM1* and *PKM2*, which action occurred through direct targeting of *PKM* to enable the expression of *PKLR*. Moreover, the expression levels of these miRNAs were downregulated in cancer cells that had originated from these tissues, resulting in *PKM2* dominance. Our results suggest that the organ-specific distribution of miRNAs is one of the principal means by which miRNA establishes characteristics of a tissue and that dysregulation of these miRNAs results in cancer development through a change in the ratio of *PKM* isoform expression. Also, our results contribute to cancer diagnosis and will be useful for cancer-specific therapy for the Warburg effect in the near future.

## 1. Introduction

Pyruvate kinase (PK) is known as the glycolytic enzyme catalyzing the final step in glycolysis. In mammals, two different PK enzymes exist, i.e., pyruvate kinase M 1/2 (*PKM*) and pyruvate kinase L/R (*PKLR*). Also, *PKM* has two isoforms, i.e., *PKM1*/*PKM2*, and they are encoded by the M gene [1]. Alternative splicers, such as those in the heterogeneous nuclear ribonucleoprotein (HNRNP) family, regulate the expression of *PKM1* and *PKM2* isoforms through splicing of exon 9 and exon 10 [2]. Namely, the adoption of exon 9 induces *PKM1*; and that of exon 10, *PKM2*. Among HNRNP family members, polypyrimidine tract binding protein 1 (*PTBP1*) is one of the crucial splicers for *PKM2*-dominant expression, acting by repressing the expression of exon 9 [3]. Recently, *PKM2* has been recognized as one of the crucial oncogenes in various cancer cells. In other words, *PKM2* contributes to the establishment of the cancer-specific energy metabolism termed the “Warburg effect” [4]. Several reports have suggested that upregulation of *PKM2* induces reprogramming of the Warburg effect in cancer cells. For example, *PKM2* promotes aerobic glycolysis in order to acquire an advantageous environment of tumor cells by the production of lactic acid and nucleic acid [4,5]. Also, *PKM2* has multiple functions for cancer growth, such as transcriptional regulation and extracellular signaling [6]. It has been thought that the *PKM* isoform switches from *PKM1* to *PKM2* to establish the Warburg effect during carcinogenesis [7]. However, our group and another have uncovered new features about this switching machinery. Namely, *PKM1* is predominant only in brain, skeletal muscle, and heart among adult tissues. In these tissues, *PKM* switching machinery causes an expression change from *PKM1* to *PKM2* during cancer development. However, in other proliferative tissues, such as colon and stomach, *PKM2* exists even in normal tissue, and the *PKM2* ratio is further increased during cancer development [8,9].

On the other hand, *PKLR* is encoded by the L gene [10]. *PKLR* has two isoforms, i.e., *PKL* and *PKR*. *PKL* is predominant in the liver, and *PKR* in red blood cells (RBCs) [4]. *PKLR* acts as a feed-forward regulator of glycolysis [4,11]. Mutations in the *PKLR* gene cause severe congenital disease, such as nonspherocytic hemolytic anemia [12]. Thus, *PKM* and *PKLR* are clearly distributed in a tissue-specific manner. However, the mechanisms underlying their tissue distribution have been largely unknown.

MicroRNA (miRNA, *MIR*) negatively regulates the expression of its target gene(s) through repression of translation or induction of degradation of its target mRNA(s). MiRNAs are associated with various phenomena, including tissue differentiation [13] and carcinogenesis [14]. Individual miRNAs function as fine adjusters of gene expression, and many miRNAs modulate a given phenomenon by acting together. Recently, we found that some organ-specific miRNAs, such as brain-specific *MIR124* and muscle-specific *MIR-133b*, determine *PKM* isoform expression through direct targeting of *PTBP1* [8]. Also, the dysregulated expression of these miRNAs contributes to carcinogenesis through the modulation of the Warburg effect in gastrointestinal tumors [15,16]. We have assumed that some organ-specific miRNAs contribute strongly to characteristic functions and carcinogenesis of their tissue. To confirm our hypothesis, we needed to examine whether this machinery was operating even in the case of other miRNAs. Hence, in this study, we verified the universality of our hypothesis by using other *PTBP1*- and *PKM*-targeting miRNAs.

## 2. Results

### 2.1. Expression of Pyruvate Kinase (PK) Enzymes Revealed Organ Specificity

**Figure 1:**
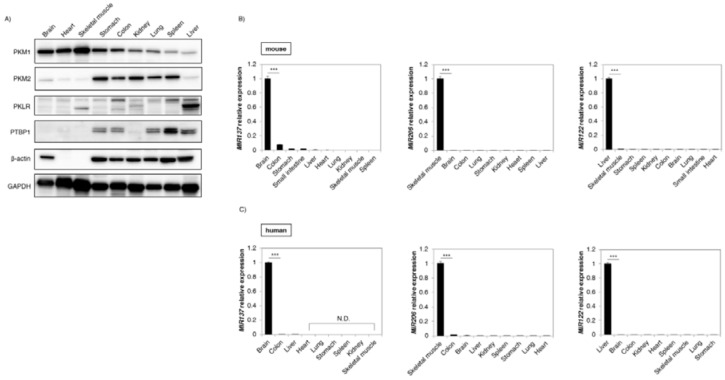
Pyruvate kinase (PK) different forms and PK enzymes regulating miRNAs showed organ specificity. (**A**) Expression profile of *PKM1*, *PKM2*, pyruvate kinase L/R (*PKLR*), and *PTBP1* in tissues from mouse organs. β-actin and glyceraldehyde-3-phosphate dehydrogenase (*GAPDH*) were used as internal controls; (**B**) Expression profile of *MIR*137 (**Left** panel), *MIR206* (**Middle** panel), and *MIR122* (**Right** panel) in tissues from mouse organs; (**C**) Profiles of the same miRNAs in tissues from human organs. The expression level is presented as a relative value compared with the tissue that had the highest expression level in each miRNA. Notably, normalization was not performed in this case because the organ specificities of these miRNAs were strong enough so as not to be affected by normalization. Human brain cortex and liver were obtained from Biochain, and others from Clontech. Results are presented as the mean ± standard deviation (SD); *** *p* < 0.001; N.D., not detected.

Firstly, we examined the tissue distribution of PK enzymes in the tissues from mouse organs. As shown in Figure 1A, the protein expression level of *PKM1* was dominant in brain, heart, and skeletal muscle, whereas that of *PKM2* expression was relatively greater in stomach, colon, kidney, lung, and spleen tissue. In the liver, *PKM1* and *PKM2* protein levels were not noticeably expressed as compared to the abundant expression of *PKLR*. Moreover, *PTBP1*, which is a crucial splicer for *PKM2*-dominant expression in *PKM* gene splicing, was expressed in the *PKM2*-dominant tissues (except kidney and liver). These findings showed that the expression profiles of PK enzymes indicated organ specificity and that *PTBP1* might be associated with this specificity.

### 2.2. Expression of MicroRNA (MIR) 137, 206, and 122 Was Organ-Specific

Some organ-specific miRNAs, such as *MIR1*, *124*, and *133b*, which directly target *PTBP1*, regulate *PKM1* and *PKM2* expression [8,15]. The Target Scan 7.1 database (http://www.targetscan.org/) showed that *MIR137* and *206* had a site that binds directly to the 3′UTR of *PTBP1* and that only *MIR122* had one directly binding to the 3′UTR of *PKM* [17]. Also, we validated the association of each miRNA and targeting gene with miRTarBase (http://mirtarbase.mbc.nctu.edu.tw/php/index.php) (Table 1). Next, we investigated the tissue distribution of these miRNAs in the tissues from mouse organs. As shown in Figure 1B, *MIR*137 was considerably and dominantly distributed in the brain, with *MIR206* being expressed in skeletal muscle. Also, *MIR122* was considerably dominant in the liver. Moreover, these findings were similar to those obtained for human tissues (Figure 1C). These findings suggested that such organ-specific expression may be associated with the *PKM* expression profile.

### 2.3. MIR137 and 206 Directly Bound to Polypyrimidine Tract Binding Protein 1 (PTBP1) and Regulated Expression of the Pyruvate Kinase M 1/2 (PKM) Isoform

**Figure 2:**
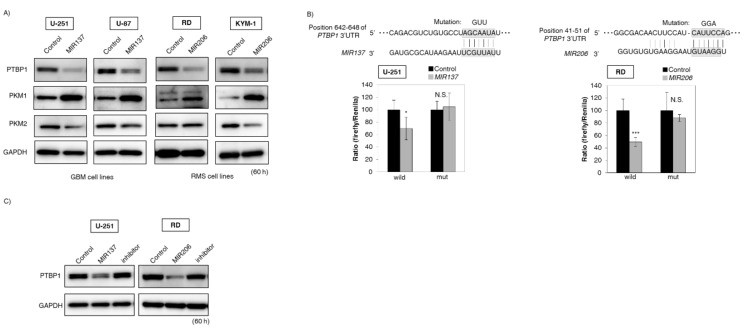

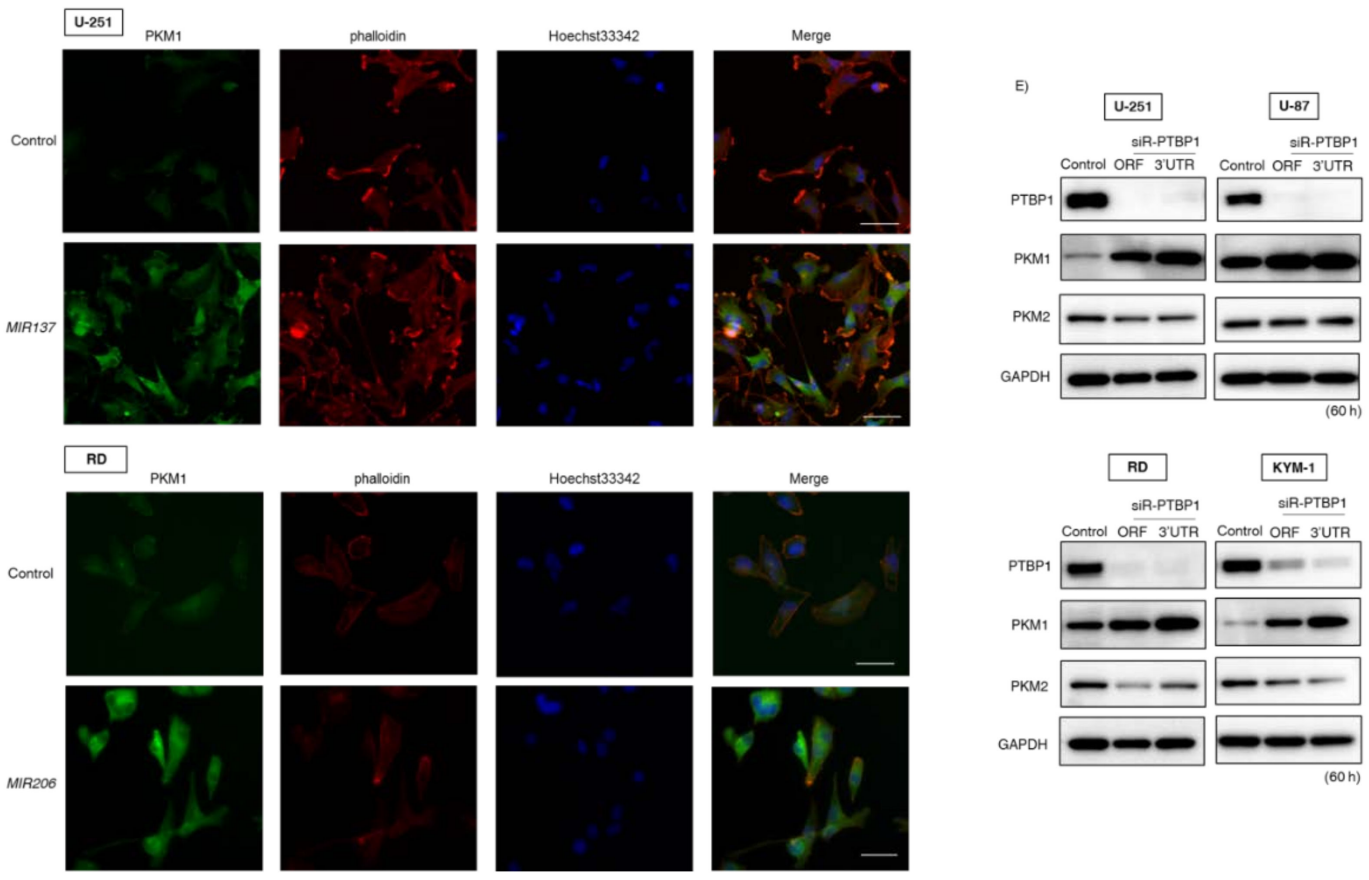
*MIR137* and *206* regulated *PKM* isoform expression through the direct targeting of *PTBP1* mRNA. (**A**) Protein expression levels of *PTBP1*, *PKM1*, and *PKM2* at 60 h after the transfection with *MIR137* or *206*. Human glioblastoma (GBM) cell lines (U-251 and U-87) were transfected with brain-specific *MIR137* (20 nM). Also, rhabdomyosarcoma (RMS) cell lines (RD and KYM-1) were transfected with muscle-specific *MIR206* (20 nM); (**B**) Luciferase activities after co-transfection with control or *MIR137* or *206* and wild-type or mutant-type pMIR vectors having the predicted *MIR137* or *206* binding site in the 3′UTR of *PTBP1*. The upper panel shows the region of the 3′ UTR of human *PTBP1* mRNA complementary to the mature *MIR137* or *206*. The box indicates the predicted binding sites for *MIR137* or *206*. The left panel is the case of *MIR137* transfected with U-251 cells. The right panel is the case of *MIR206* transfected with RD cells. Effects were assessed at 24 h after the transfection (20 nM); (**C**) Protein expression levels of *PTBP1* in U-251 and RD cells after combined treatment with antago*MIR137*/*MIR137* or antago*MIR206*/*MIR206*. Control lane: treatment with control miRNA (20 nM). *MIR137* or *206* lane: treatment with *MIR137* or *206* (20 nM). Inhibitor lane: treatment with *MIR137* or *206* (10 nM) + antago*MIR137* or *206* (10 nM). Effects were assessed at 60 h after the transfection; (**D**) Immunofluorescence of *PKM1* at 60 h after transfection of U-251 cells with *MIR137* or transfection of RD cells with *MIR206*. **Upper** panels, treatment with control miRNA (20 nM); **Lower** panels, treatment with *MIR137* or *206* (20 nM). *PKM1* is dyed green; cell membrane, red; and nuclei, blue. Representative photographs from two experiments are shown. Scale bar = 50 µm; (**E**) Protein expression levels of the *PKM* isoform in GBM and RMS cells at 60 h after siR-*PTBP1* transfection (5 nM). ORF: open reading frame. Results are presented as the mean ± SD; * *p* < 0.05, *** *p* < 0.001; N.S., not statistically significant.

In order to further clarify the relationship between the expression of the miRNAs and *PTBP1*, we examined whether brain-specific *MIR137* and muscle-specific *MIR206* could directly bind to *PTBP1*. We selected glioblastoma (GBM) cell lines, i.e., U-251 and U-87, in the case of brain-specific *MIR137* and rhabdomyosarcoma (RMS) RD and KYM-1 cell lines in the case of muscle-specific *MIR206*. As a result, the protein expression levels of *PTBP1* were downregulated in these miRNA-treated cells tested. Also, the expression level of *PKM1* was increased and that of *PKM2* was decreased as expected (Figure 2A). The luciferase reporter activity of wild-type pMIR-*PTBP1* was significantly inhibited after the introduction of brain-specific *MIR137* into U-251 cells or muscle-specific *MIR206* into RD cells. On the other hand, mutation of the *PTBP1* 3′ UTR-binding site abolished the inhibitory effect of these miRNAs (Figure 2B). In addition, treatment with an inhibitor of these miRNAs reversed the expression level of *PTBP1* in both U-251 and RD cells (Figure 2C). Moreover, immunofluorescence (IFC) indicated increased signal intensity of *PKM1* in these miRNAs-treated cells (Figure 2D). Furthermore, Western blotting analysis showed that knockdown of *PTBP1* gave similar results; i.e., an increase in the *PKM1*/*PKM2* ratio (by switching from *PKM2* to *PKM1*) was observed in all cells tested (Figure 2E). These findings suggested that these organ-specific miRNAs directly bound to *PTBP1* mRNA and regulated the expression of the *PKM* isoform.

### 2.4. MIR122 Directly Bound to PKM and Negatively Regulated PKM2 Expression

**Figure 3:**
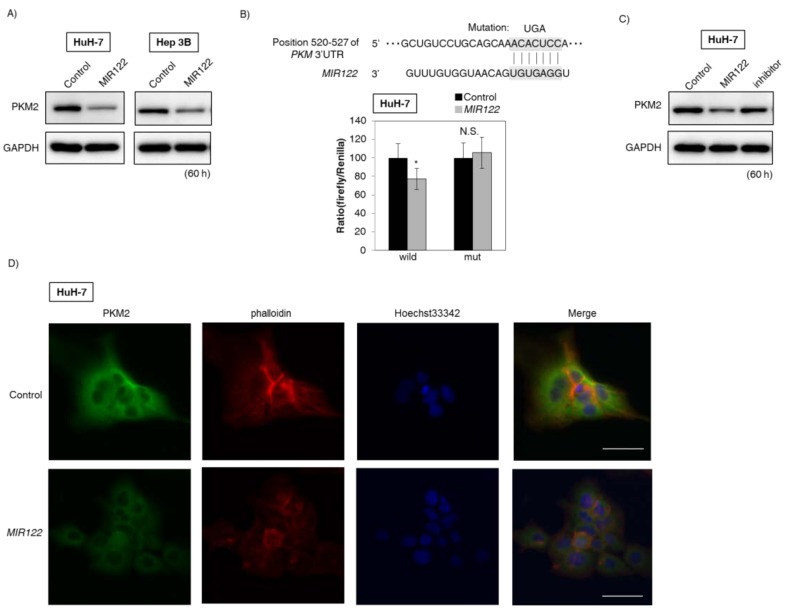
*MIR122* negatively regulated *PKM* expression through the direct targeting of *PKM* mRNA. (**A**) Protein expression levels of *PKM2* at 60 h after the transfection of hepatocellular carcinoma (HCC) cell lines (HuH-7 and Hep 3B) with *MIR122* (20 nM); (**B**) Luciferase activities after co-transfection with control or *MIR122* and wild-type or mutant-type pMIR vectors having the predicted *MIR122* binding site in the 3′-UTR of *PKM*. The upper panel shows the region of the 3′UTR of human *PKM* mRNA complementary to the mature *MIR122*. The box indicates the predicted binding sites for *MIR122*. Effects were assessed at 24 h after the transfection (20 nM); (**C**) Protein expression levels of *PKM2* in HuH-7 cells after combined treatment with antago*MIR122*/*MIR122*. Control lane: treatment with control miRNA (20 nM). *MIR122* lane: treatment with *MIR122* (20 nM). Inhibitor lane: treatment with *MIR122* (10 nM) + antago*MIR122* (10 nM). Effects were assessed at 60 h after the transfection; (**D**) Immunofluorescence of *PKM2* at 60 h after transfection of HuH-7 cells with *MIR122* (20 nM). **Upper** panel, treatment with control miRNA; **Lower** panel, treatment with *MIR122*. *PKM2* is dyed green; cell membrane, red; and nuclei, blue. Representative photographs from two experiments are shown. Scale bar = 50 µm. Results are presented as the mean ± SD; * *p* < 0.05; N.S., not statistically significant.

Next, we examined whether liver-specific *MIR122* would directly bind to *PKM*. We selected hepatocellular carcinoma (HCC) cell lines for these experiments. As shown in Figure 3A, protein expression levels of *PKM2* were downregulated in *MIR122*-treated HCC cells. In addition, the luciferase reporter activity of wild-type pMIR-*PKM* was significantly inhibited after the introduction of liver-specific *MIR122* into HuH-7 cells. On the other hand, mutation of the *PKM* 3′UTR-binding site abolished the inhibitory effect of liver-specific *MIR122* (Figure 3B). Moreover, treatment with an inhibitor of *MIR122* reversed the expression level of *PKM2* in HuH-7 cells (Figure 3C). In addition, IFC indicated that the signal intensity of *PKM2* was decreased in the *MIR122*-treated HuH-7 cells (Figure 3D). These findings suggested that liver-specific *MIR122* directly bound to *PKM* mRNA and negatively regulated *PKM2* expression.

### 2.5. Dysregulation of These Organ-Specific miRNAs Contributed to Carcinogenesis through the Modulation of Expression of Warburg-Effect-Associated Genes

**Figure 4:**
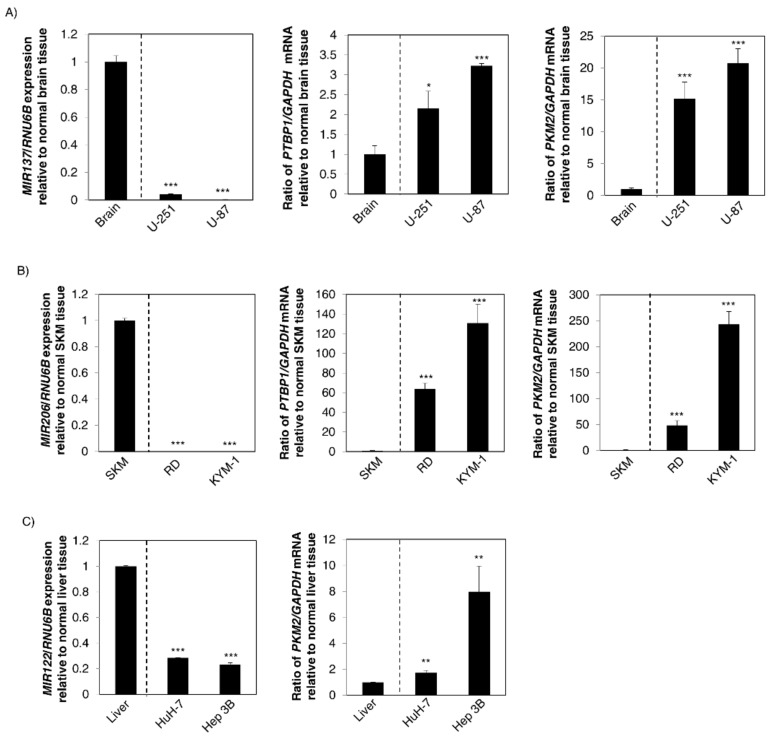
Downregulation of these organ-specific miRNAs contributed to carcinogenesis through upregulation of Warburg-effect-related genes. (**A**) **Left** panel: Expression levels of *MIR137* in the normal human brain tissues and GBM cell lines; **Middle** panel: mRNA expression levels of *PTBP1* in the same samples; **Right** panel: That of *PKM2* mRNA expression; (**B**) **Left** panel: Expression levels of *MIR206* in the normal human skeletal muscle tissues and RMS cell lines; **Middle** panel: The mRNA expression levels of *PTBP1* in the same samples; **Right** panel: That of *PKM2* mRNA expression; SKM: skeletal muscle; (**C**) **Left** panel: Expression levels of*MIR122* in the normal human liver tissues and HCC cell lines; **Right** panel: mRNA expression levels of *PKM2* in the same samples. Results are presented as the mean ± SD; * *p* < 0.05; ** *p* < 0.01; *** *p* < 0.001.

**Figure 5:**
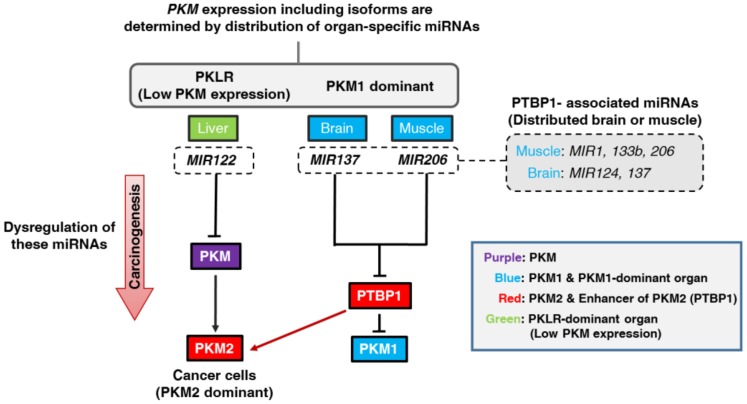
Schematic diagram of our study findings. *PKLR* and *PKM* isoforms showed organ specificity; i.e., *PKLR* was dominantly expressed in the liver. *PKM1* was dominantly expressed in the brain and muscle. In the liver, liver-specific *MIR122* suppressed the *PKM* expression by dominant distribution of *MIR122*. In the brain and muscle, brain-specific *MIR137* and muscle-specific *MIR206* induced the upregulation of the *PKM1* expression through the suppression of the *PTBP1* expression. Previously, we proved that muscle-specific *MIR1* and *133b* and brain-specific *MIR124* had the same mechanism for *PKM* isoform expression. These organ-specific miRNAs, which targeted *PTBP1* directly, regulate *PKM* isoform expression by their tissue distribution. We named these miRNAs *PTBP1*-associated miRNAs. Also, dysregulation of these organ-specific miRNAs strongly contributed to carcinogenesis by promoting *PKM2* expression in cancer cells (Red arrow). Colors indicate the following: Purple; *PKM* gene, Blue; *PKM1* and *PKM1*-dominant organ, Red; *PKM2* and enhancer of *PKM2* (*PTBP1*), Green; *PKLR-*dominant (low *PKM* expression) organ.

To clarify the importance of these miRNAs in carcinogenesis, we examined the association between the expression levels of these organ-specific miRNAs and the Warburg-effect-related genes, i.e., *PTBP1* and *PKM2*. As shown in Figure 4A, brain-specific *MIR137* was downregulated in GBM cell lines compared with its expression in the normal brain tissue; and mRNA expression levels of *PTBP1* and *PKM2* were upregulated. For *MIR206*, similar findings were also observed for the RMS cell lines tested (Figure 4B). On the other hand, liver-specific *MIR122* was downregulated in HCC cell lines compared with its expression in normal liver tissue; and the mRNA expression level of *PKM2* was increased (Figure 4C). These findings suggested that dysregulation of these organ-specific miRNAs was strongly associated with the Warburg effect through the change in *PKM2* expression (Figure 5).

## 3. Discussion

In this study, we showed that brain-specific *MIR137* and muscle-specific *MIR206* enabled *PKM1*-dominant expression predominantly in brain and skeletal muscle tissues through the direct targeting of *PTBP1* mRNA (Figure 2). Recently, we indicated that brain-specific *MIR124* and muscle-specific *MIR1* and *133b* have the same machinery for the processing of *PKM* isoform expression [8,19]. Moreover, presently we showed that liver-specific *MIR122* suppressed both *PKM1* and *PKM2* expression in normal liver tissue by binding to the 3′UTR of *PKM* (Figure 3). Based on these findings, we concluded that levels of PK enzymes, especially in the case of *PKM* expression, were strongly determined by organ-specific miRNAs. This is one of the most typical patterns of the miRNA system to determine tissue characteristics. Such organ specificities would have some biological significance for the fate or commitment of tissue differentiation. Of course, further investigations are required, because all of the miRNAs that regulate *PTBP1* expression, such as *MIR9*, *153*, and *200bc*, were not examined in our studies. The association between these organ-specific miRNAs and carcinogenesis is extremely important. For example, it was reported earlier that *MIR124* and *137*, which are predominant in the brain, are frequently downregulated in brain tumors, such as GBM [20], and that downregulation of *MIR122* induces HCC carcinogenesis [21]. Similarly, dysregulation of muscle-specific miRNAs, such as *MIR1*, *133* and *206*, contribute to the development of sarcomas, including RMS [19,22]. Furthermore, we indicated that dysregulated expression of *MIR145*, which is predominant in the gastrointestinal tract, contributes significantly to the development of gastrointestinal cancer [23,24]. Namely, dysregulation of miRNAs, whose normal expression enables an organ to acquire its characteristics, most deeply contributes to carcinogenesis in these tissues. Hence, we should focus on dysregulation of these organ-specific miRNAs to better understand the mechanisms of organ-specific carcinogenesis.

Our recent study indicated that *PTBP1* functions as a novel oncogene and that *PTBP1* promotes the Warburg effect in various types of cancer [15,16,25]. Previously, *PTBP1* was recognized as an oncogene in GBM [3,26] and as having an onco-genetic function in breast cancer cell lines and ovarian tumors [27,28]. It has been reported that *c-Myc* is a positive regulator of *PTBP1* [3]. However, we found earlier that various organ-specific miRNAs negatively regulate *PTBP1* expression both directly and indirectly [8,16,29]. The induction of switching of a *PKM* isoform from *PKM2* to *PKM1* in various cancer cells by ectopic expression of these miRNAs supports our opinion [15,18,30]. Of course, other splicers, such as *HNRNPA1* and Serine and argininerich splicing factor 3 (*SRSF3*), work jointly with *PTBP1* in the process of *PKM* isoform expression [3,31]. Actually, in the case of HCC cell lines, the increase in *PKM2* expression in HuH-7 cells was relatively less than that of it in Hep 3B cells compared with the decrease in miR-122 expression (Figure 4C). Hence, further investigation of the association between other such splicers of the *PKM* gene and miRNAs will more fully clarify the mechanisms of *PKM* isoform expression. Understanding of the mechanism underlying the maintenance of *PKM* isoform expression is essential for clarifying the mechanism of carcinogenesis. Also, such understanding will contribute to clarifying the conditions for advanced-stage cancer, such as metastasis [32]. We strongly believe that our results contribute to cancer diagnosis and will be useful for cancer-specific therapy for the Warburg effect in the near future.

## 4. Materials and Methods

### 4.1. miRNA Validation and Terminology

Firstly, the Target Scan 7.1 database (http://www.targetscan.org/) was used in order to find the binding sites of each miRNA and targeting gene [17]. Also, searching in more detail for associations between them was performed with miRTarBase (http://mirtarbase.mbc.nctu.edu.tw/php/index.php). The validations are summarized in Table 1. The terminology of miRNAs was based on miRNA nomenclature guidelines [33,34].

### 4.2. Samples from Human and Mouse

Human RNA samples were obtained from Clontech (TAKARA BIO INC., Shiga, Japan) or Biochain (BioChain Institute Inc., Newark, CA, USA). BALB/cSLC-nu/nu (nude) mice were obtained from Japan SLC (Shizuoka, Japan). Animal experimental protocols were approved by the Committee for Animal Research and Welfare of Gifu University (11021, 10 February 2016).

### 4.3. Cell Culture

Human glioblastoma (GBM) cell lines U-251 and U-87, those of rhabdomyosarcoma (RMS) cell line RD and KYM-1, and those of hepatocellular carcinoma (HCC) cell line HuH-7 and Hep 3B were used in this study. Human rhabdomyosarcoma cell lines RD and KYM-1 and human hepatocellular carcinoma cell line HuH-7 were obtained from JCRB (Japanese Collection of Research Bioresources) Cell Bank. Human hepatocellular carcinoma cell line Hep 3B was a gift from Nobuhiko Tanigawa (an Emeritus Professor of our department). U-251 and U-87 were provided by Atsushi Natsume (Department of Neurosurgery Nagoya University, Graduate School of Medicine). U-251, U-87, HuH-7, and Hep B3 cells were cultured in DMEM medium. RD cells were grown in Eagle’s minimal essential medium, and KYM-1 cells in a mixture of Ham’s F12 medium and Dulbecco’s modified Eagle’s medium (1:1). Ten percent (*v*/*v*) heat-inactivated FBS (Sigma-Aldrich, St. Louis, MO, USA) and 2 mM l-glutamine were supplied to each medium. The temperature and atmosphere were maintained at 37 °C and 95% air with 5% CO_2_, respectively.

### 4.4. Western Blotting

Cells of each type were homogenized in cold-lysis buffer. The composition of it was given in previous reports of ours [8,35]. Protease Inhibitor Cocktail (1%) (Sigma-Aldrich) was added to it and reacted for 15 min on ice. Centrifugation (13,000 rpm for 20 min at 4 °C) was performed and the supernatants were collected. Protein contents were calculated with a DC Protein assay kit (Bio-Rad Laboratories, Inc., Hercules, CA, USA). Six to 10 micrograms of samples were separated by SDS-PAGE using appropriate polyacrylamide gels (10.0% or 12.5%; Wako Pure Chemical Industries, Ltd., Osaka, Japan). Also, separated samples were electro-blotted onto a PVDF membrane (PerkinElmer Life Sciences, Inc., MA, USA or Bio-Rad Laboratories, Inc.). The blockage of nonspecific binding sites was performed with 5% nonfat milk (Cell Signaling Technology, Inc., Danvers, MA, USA) in PBS containing 0.1% Tween 20 (TBS-T). The membrane was incubated for the appropriate time (several hours to 1 day) at 4 °C with primary antibodies. After that, the membrane was washed with TBS-T, after which secondary antibodies were added. The immunoreaction proceeded over a 1-h period at room temperature. Finally, the immunoblots were visualized with Luminata^TM^ Forte Western HRP Substrate (Millipore Corporation, Billerica, MA, USA). We detected protein expression by using an Image Quant Luminescent Image Analyzer LAS-4000 (Fuji Photo Film Co., Tokyo, Japan) or Fusion-FX7 (Vilber Lourmat, Marne-la-Vallée, France). Primary antibodies used were as follows: anti-*PKM1*, -*PKM2*, -*PTBP1*, -β-actin, and -GAPDH (Cell Signaling Technology, Inc., Danvers, MA, USA) and anti-*PKLR* (GeneTex, Inc., Irvine, CA, USA). Secondary antibodies (HRP-conjugated goat anti-rabbit and horse anti-mouse IgG) were obtained from Cell Signaling Technology. GAPDH or β-actin was used as an internal control according to our previous report [8].

### 4.5. Real-Time Reverse Transcription-PCR

MiRNA was isolated from cultured cells by using a NucleoSpin microRNA isolation kit (Takara Bio Inc., Shiga, Japan). The relative expression levels were calculated by the ΔΔ*C*_t_ method. Each value of ΔΔ*C*_t_ was determined by use of a Thermal Cycler Dice Real Time System II model TP900/960 or TP870 (Takara Bio Inc.). The primers for *PTBP1*, *PKM1*, *PKM2*, and *GAPDH* were the following: *PTBP1*-sense, 5′-ATCAGGCCTTCATCGAGATGCACA-3′, and *PTBP1*-antisense, 5′-TGTCTTGAGCTCCTTGTGGTTGGA-3′; *PKM1*-sense, 5′-CGAGCCTCAAGTCACTCCAC-3′, and *PKM1*-antisense, 5′-GTGAGCAGACCTGCCAGACT-3′; *PKM2*-sense, 5′-ATTATTTGAGGAACTCCGCCGCCT-3′, and *PKM2*-antisense, 5′-ATTCCGGGTCACAGCAATGATGG-3′; *GAPDH*-sense, 5′-CCACCCATGGCAAATTCCATGGCA-3′, and *GAPDH*-antisense, 5′-TCTAGACGGCAGGTCAGGTCCACC-3′. *GAPDH* and *RNU6B* were used as internal controls. Normalization was not performed in the case of expression profiles of organ-specific miRNAs.

### 4.6. Transfection Experiments

All cells were seeded in 6-well plates at a concentration of 0.5 × 10^5^ per well (10–30% confluence) on the day before the transfection. Each transfection was performed by using Lipofectamine^TM^ RNAiMAX (Invitrogen, Carlsbad, CA, USA) according to the manufacturer’s protocol. The mature types of *MIR122*, *137*, and *206* (mirVanaTM miRNA mimic; Ambion, CA, USA), antago*MIR122*, *137*, and *206* (mirVana^TM^ miRNA inhibitor; Ambion), or siRNA for *PTBP1* (siR-*PTBP1*; Invitrogen, Carlsbad, CA, USA) were used for the transfection of the cells. The sequence of each miRNA or siRNA was as indicated below. The sequence of the mature type of *MIR122* used in this study was 5′-UGGAGUGUGACAAUGGUGUUUG-3′; that of *MIR137*, 5′-UUAUUGCUUAAGAAUACGCGUAG-3′; that of *MIR206*, 5′-UGGAAUGUAAGGAAGUGUGUGG-3′; that of siR-*PTBP1* for the open reading frame region, 5′-UGUCAUUUCCGUUUGCUGCAGAAGC-3′; and that of the 3′UTR region, 5′-AUCUCUGGUCUGCUAAGGUCACUUC-3′. The nonspecific miRNA (HSS, Hokkaido, Japan) sequence of 5′-GUAGGAGUAGUGAAAGGCC-3′ was used as a control for nonspecific effects [25,36].

### 4.7. Luciferase Reporter Assay

By searching the Target Scan 7.1 database (http://www.targetscan.org/) to find algorithm-based binding sites of *MIR137* or *206*, we found the predicted binding site to be at position 642–648 for *MIR137* and at 45–51 for *MIR206* in the 3′UTR of *PTBP1* mRNA [17]. Also, we found the predicted binding site to be at position 520–527 for *MIR122* in the 3′UTR of *PKM* mRNA. The sequence region containing the putative binding sequence of *MIR137*, *206*, or *122* was inserted into a pMIR-REPORT^TM^ Luciferase miRNA Expression Reporter Vector (Applied Biosystems Inc., Foster City, CA, USA) according to the manufacturer’s protocol. Moreover, we made other pMIR constructs, one encompassing a mutated seed sequence for *MIR137* (wild-type, AGCAAUA; mutant, AGGUUUA), another for *206* (wild-type, CAUUCCAG; mutant, CAGGACAG), and another for *122* (wild-type, ACACUCC; mutant, ACUGACC) by using a PrimeSTAR^®^ Mutagenesis Basal Kit (Takara Bio Inc.). The mutation of each vector was confirmed by sequence analysis. A pRL-TK Renilla Luciferase Reporter vector (Promega Corporation, Madison, WI, USA) was used as an internal control vector. Cells were seeded into 96-well plates at a concentration of 0.1 × 10^4^ per well on the day before the transfection. Each cell type was co-transfected with a reporter vector (wild-type or mutated type; 0.01 μg/well) and *MIR137*, *206*, *122*, or a nonspecific non-coding siRNA (Dharmacon, Tokyo, Japan). Luciferase activities were measured by using a Dual-Glo Luciferase Assay System (Promega Corporation, Madison, WI, USA). These activities were calculated as the ratios of firefly luciferase/Renilla luciferase to define luciferase activity as in the case of our previous studies [15].

### 4.8. Immunofluorescence (IFC) Study

Cells of each cell type were seeded into the wells of a Lab-Tek II Chamber Slide System (Thermo Fisher Scientific Inc., Waltham, MA, USA). After transfection with a given miRNA including nonspecific miRNA (Control miRNA), the cells were immunostained with anti-*PKM1* or -*PKM2* antibody according to the immunofluorescence protocol of Cell Signaling Technology. The nuclei were stained with Hoechet33342 (Sigma-Aldrich), and for actin labeling the cells were incubated with a fluorescent F-actin probe, Rhodamine Phalloidin (Cytoskeleton Inc., Denver, CO, USA). The cells were observed with a BIOREVO fluorescence microscope (Keyence, Osaka, Japan).

### 4.9. Statistics

The statistical examinations were performed in triplicate. The two-sided Student’s *t*-test was used for determining the statistical significance of differences. The mean ± standard deviation was indicated in necessary cases. Statistical significance was set at a *p*-value < 0.05.

## Figures and Tables

**Table 1:** Validations of microRNAs (miRNAs) used in this study.

**miRNA gene name (ID)**	*MIR122* (406906)	*MIR137* (406928)	*MIR206* (406989)
**Target gene name (ID)**	*PKM* (5315)	*PTBP1* (5725)	*PTBP1* (5725)
**Species name (ID)**	*Homo sapiens* (9606)	*Homo sapiens* (9606)	*Homo sapiens* (9606)
**Genomic location of MTI** **Nucleotide sequence**	5′ACACTCC	5′AGCAATA	5′CATTCCA
**Location**	15:72199124–72199130	19:811468–811474	19:810871–810876
**Location within a part of a gene**	520–527 (location within 3′UTR)	642–648 (location within 3′UTR)	45–51 (location within 3′UTR)
**Existence of previous report** **(searching with miRTarBase)**	Yes	No [18]	No
**Methods for experimental validation in this study**	Luciferase reporter assay Western blot analysis	Luciferase reporter assay Western blot analysis	Luciferase reporter assay Western blot analysis
**Experimental materials used in this study**	HuH-7 and Hep 3B cell lines	U-251 and U-87 cell lines	RD and KYM-1 cell lines

*PKM* = pyruvate kinase M1/2; *PTBP1 =* polypyrimidine tract binding protein 1; 3′UTR = 3′ untranslated region; *MIR = microRNA (gene symbol)*; HuH-7 and Hep 3B = human hepatocellular carcinoma cell lines; U-251 and U-87 = human glioblastoma cell lines; RD and KYM-1 = human rhabdomyosarcoma cell lines; MTI = microRNA-target interaction.

## Abbreviations

GAPDH	glyceraldehyde-3-phosphate dehydrogenase
GBM	glioblastoma
HCC	hepatocellular carcinoma
HNRNP	heterogeneous nuclear ribonucleoproteins
IFC	immunofluorescence
*MIR*	microRNA (gene symbol in human)
miRNA	microRNA
MTI	microRNA-target interaction
ORF	open reading frame
*PKLR*	pyruvate kinase L/R
*PKM*	pyruvate kinase M1/2
*PTBP1*	polypyrimidine tract binding protein 1
RMS	rhabdomyosarcoma
SRSF3	Serine and arginine-rich splicing factor 3
3′UTR	3′ untranslated region

## References

[1] Noguchi T., Inoue H., Tanaka T. (1986). The M1- and M2-type isozymes of rat pyruvate kinase are produced from the same gene by alternative RNA splicing. J. Biol. Chem..

[2] Clower C.V., Chatterjee D., Wang Z., Cantley L.C., Heiden M.G.V., Krainer A.R. (2010). The alternative splicing repressors hnRNP A1/A2 and PTB influence pyruvate kinase isoform expression and cell metabolism. Proc. Natl. Acad. Sci. USA.

[3] David C.J., Chen M., Assanah M., Canoll P., Manley J.L. (2010). HnRNP proteins controlled by c-Myc deregulate pyruvate kinase mRNA splicing in cancer. Nature.

[4] Dayton T.L., Jacks T., Heiden M.G.V. (2016). PKM2, cancer metabolism, and the road ahead. EMBO Rep..

[5] He X., Du S., Lei T., Li X., Liu Y., Wang H., Tong R., Wang Y. (2017). PKM2 in carcinogenesis and oncotherapy. Oncotarget.

[6] Hsu M.C., Hung W.C. (2018). Pyruvate kinase M2 fuels multiple aspects of cancer cells: From cellular metabolism, transcriptional regulation to extracellular signaling. Mol. Cancer.

[7] Chen M., Zhang J., Manley J.L. (2010). Turning on a fuel switch of cancer: HnRNP proteins regulate alternative splicing of pyruvate kinase mRNA. Cancer Res..

[8] Taniguchi K., Ito Y., Sugito N., Kumazaki M., Shinohara H., Yamada N., Nakagawa Y., Sugiyama T., Futamura M., Otsuki Y. (2015). Organ-specific PTB1-associated microRNAs determine expression of pyruvate kinase isoforms. Sci. Rep..

[9] Bluemlein K., Gruning N.M., Feichtinger R.G., Lehrach H., Kofler B., Ralser M. (2011). No evidence for a shift in pyruvate kinase PKM1 to PKM2 expression during tumorigenesis. Oncotarget.

[10] Noguchi T., Yamada K., Inoue H., Matsuda T., Tanaka T. (1987). The L- and R-type isozymes of rat pyruvate kinase are produced from a single gene by use of different promoters. J. Biol. Chem..

[11] Dombrauckas J.D., Santarsiero B.D., Mesecar A.D. (2005). Structural basis for tumor pyruvate kinase M2 allosteric regulation and catalysis. Biochemistry.

[12] Larochelle A., Magny P., Tremblay S., Medicis E.E. (1999). Erythropoiesis: Pyruvate kinase deficiency which causes nonspherocytic hemolytic anemia: The gene and its mutations. Hematology.

[13] Zeng Z.L., Lin X.L., Tan L.L., Liu Y.M., Qu K., Wang Z. (2018). MicroRNAs: Important regulators of induced pluripotent stem cell generation and differentiation. Stem Cell Rev..

[14] Fang Y., Zhang L., Li Z., Li Y., Huang C., Lu X. (2017). MicroRNAs in DNA damage response, carcinogenesis, and chemoresistance. Int. Rev. Cell Mol. Biol..

[15] Taniguchi K., Sakai M., Sugito N., Kumazaki M., Shinohara H., Yamada N., Nakayama T., Ueda H., Nakagawa Y., Ito Y. (2016). PTBP1-associated microRNA-1 and -133b suppress the Warburg effect in colorectal tumors. Oncotarget.

[16] Sugiyama T., Taniguchi K., Matsuhashi N., Tajirika T., Futamura M., Takai T., Akao Y., Yoshida K. (2016). MiR-133b inhibits growth of human gastric cancer cells by silencing pyruvate kinase muscle-splicer polypyrimidine tract-binding protein 1. Cancer Sci..

[17] Agarwal V., Bell G.W., Nam J.W., Bartel D.P. (2015). Predicting effective microRNA target sites in mammalian mRNAs. Elife.

[18] Sugito N., Taniguchi K., Kuranaga Y., Ohishi M., Soga T., Ito Y., Miyachi M., Kikuchi K., Hosoi H., Akao Y. (2017). Cancer-specific energy metabolism in rhabdomyosarcoma cells is regulated by microRNA. Nucleic Acid Ther..

[19] Sun Y., Zhao X., Zhou Y., Hu Y. (2012). miR-124, miR-137 and miR-340 regulate colorectal cancer growth via inhibition of the Warburg effect. Oncol. Rep..

[20] Silber J., Lim D.A., Petritsch C., Persson A.I., Maunakea A.K., Yu M., Vandenberg S.R., Ginzinger D.G., James C.D., Costello J.F. (2008). miR-124 and miR-137 inhibit proliferation of glioblastoma multiforme cells and induce differentiation of brain tumor stem cells. BMC Med..

[21] Tsai W.C., Hsu S.D., Hsu C.S., Lai T.C., Chen S.J., Shen R., Huang Y., Chen H.C., Lee C.H., Tsai T.F. (2012). MicroRNA-122 plays a critical role in liver homeostasis and hepatocarcinogenesis. J. Clin. Investig..

[22] Missiaglia E., Shepherd C.J., Patel S., Thway K., Pierron G., Pritchard-Jones K., Renard M., Sciot R., Rao P., Oberlin O. (2010). MicroRNA-206 expression levels correlate with clinical behaviour of rhabdomyosarcomas. Br. J. Cancer.

[23] Akao Y., Nakagawa Y., Hirata I., Iio A., Itoh T., Kojima K., Nakashima R., Kitade Y., Naoe T. (2010). Role of anti-oncomirs miR-143 and -145 in human colorectal tumors. Cancer Gene Ther..

[24] Takagi T., Iio A., Nakagawa Y., Naoe T., Tanigawa N., Akao Y. (2009). Decreased expression of microRNA-143 and -145 in human gastric cancers. Oncology.

[25] Takai T., Yoshikawa Y., Inamoto T., Minami K., Taniguchi K., Sugito N., Kuranaga Y., Shinohara H., Kumazaki M., Tsujino T. (2017). A novel combination RNAi toward warburg effect by replacement with miR-145 and silencing of PTBP1 induces apoptotic cell death in bladder cancer cells. Int. J. Mol. Sci..

[26] Jin W., McCutcheon I.E., Fuller G.N., Huang E.S., Cote G.J. (2000). Fibroblast growth factor receptor-1 alpha-exon exclusion and polypyrimidine tract-binding protein in glioblastoma multiforme tumors. Cancer Res..

[27] He X., Arslan A.D., Ho T.T., Yuan C., Stampfer M.R., Beck W.T. (2014). Involvement of polypyrimidine tract-binding protein (PTBP1) in maintaining breast cancer cell growth and malignant properties. Oncogenesis.

[28] He X., Pool M., Darcy K.M., Lim S.B., Auersperg N., Coon J.S., Beck W.T. (2007). Knockdown of polypyrimidine tract-binding protein suppresses ovarian tumor cell growth and invasiveness in vitro. Oncogene.

[29] Minami K., Taniguchi K., Sugito N., Kuranaga Y., Inamoto T., Takahara K., Takai T., Yoshikawa Y., Kiyama S., Akao Y. (2017). MiR-145 negatively regulates Warburg effect by silencing KLF4 and PTBP1 in bladder cancer cells. Oncotarget.

[30] Taniguchi K., Sugito N., Kumazaki M., Shinohara H., Yamada N., Nakagawa Y., Ito Y., Otsuki Y., Uno B., Uchiyama K. (2015). MicroRNA-124 inhibits cancer cell growth through PTB1/PKM1/PKM2 feedback cascade in colorectal cancer. Cancer Lett..

[31] Wang Z., Chatterjee D., Jeon H.Y., Akerman M., Heiden M.G.V., Cantley L.C., Krainer A.R. (2012). Exon-centric regulation of pyruvate kinase M alternative splicing via mutually exclusive exons. J. Mol. Cell Biol..

[32] Fong M.Y., Zhou W., Liu L., Alontaga A.Y., Chandra M., Ashby J., Chow A., O’Connor S.T., Li S., Chin A.R. (2015). Breast-cancer-secreted miR-122 reprograms glucose metabolism in premetastatic niche to promote metastasis. Nat. Cell Biol..

[33] Desvignes T., Batzel P., Berezikov E., Eilbeck K., Eppig J.T., McAndrews M.S., Singer A., Postlethwait J.H. (2015). miRNA nomenclature: A view incorporating genetic origins, biosynthetic pathways, and sequence variants. Trends Genet..

[34] Piletic K., Kunej T. (2017). Minimal standards for reporting microRNA: Target interactions. OMICS.

[35] Taniguchi K., Iwatsuki A., Sugito N., Shinohara H., Kuranaga Y., Oshikawa Y., Tajirika T., Futamura M., Yoshida K., Uchiyama K. (2018). Oncogene RNA helicase DDX6 promotes the process of c-Myc expression in gastric cancer cells. Mol. Carcinog..

[36] Inamoto T., Taniguchi K., Takahara K., Iwatsuki A., Takai T., Komura K., Yoshikawa Y., Uchimoto T., Saito K., Tanda N. (2015). Intravesical administration of exogenous microRNA-145 as a therapy for mouse orthotopic human bladder cancer xenograft. Oncotarget.

